# Worldwide practices on flexible endoscope reprocessing

**DOI:** 10.1186/s13756-018-0446-6

**Published:** 2018-12-17

**Authors:** N. Kenters, E. Tartari, J. Hopman, Rehab H. El-Sokkary, M. Nagao, K. Marimuthu, M. C. Vos, ISAC working group, E. G. W. Huijskens, Andreas Voss

**Affiliations:** 1 Department of Medical Microbiology, Radboud University Medical Centre, Radboudumc, Nijmegen, the Netherlands; 2 Infection Control Programme & WHO collaborating Centre of Patient, Safety, Geneva University Hospitals and Faculty of Medicine, Geneva, Switzerland; 3 Faculty of Health Sciences, University of Malta, Msida, Malta; 4 Department of Medical Microbiology and Immunology, Faculty of Medicine, Zagazig University, Zagazig, Arab Republic of Egypt; 5 Department of Infection and Prevention, Kyoto University Hospital, Kyoto, Japan; 6 Department of Infectious Diseases, Tan Tock Seng Hospital, Singapore, Republic of Singapore; 7 National Centre for Infectious Diseases, Singapore, Republic of Singapore; 8 Department of Medical Microbiology and Infectious Diseases, Erasmus MC, Rotterdam, the Netherlands; 9 International Society for Antimicrobial Chemotherapy (ISAC), IPC working group, London, England; 10 Department of Medical Microbiology, Albert Schweitzer hospital, Dordrecht, the Netherlands; 11 Department of Medical Microbiology, Canisius Wilhelmina Hospital, Nijmegen, the Netherlands

**Keywords:** Flexible endoscopes, Reprocessing, Monitoring, AER, Guidelines

## Abstract

**Background:**

Endoscopy related infections represent an important threat for healthcare systems worldwide. Recent outbreaks of infections with multidrug resistant micro-organisms have highlighted the problems of contaminated endoscopes. Endoscopes at highest risk for contamination have intricate mechanisms, multiple internal channels and narrow lumens that are especially problematic to clean. In light of raised awareness about the necessity for meticulous reprocessing of all types of endoscopes, a call for international collaboration is needed. An overview is presented on current practices for endoscope reprocessing in facilities worldwide.

**Method:**

An electronic survey was developed and disseminated by the International Society for Antimicrobials and Chemotherapy. The survey consisted of 50 questions aimed at assessing the reprocessing of flexible endoscopes internationally. It covered three core elements: stakeholder involvement, assessment of perceived risks, and reprocessing process.

**Results:**

The survey received a total of 165 completed responses from 39 countries. It is evident that most facilities, 82% (*n* = 136), have a standard operating procedure. There is, however a lot of variation within the flexible endoscope reprocessing practices observed. The need for regular training and education of reprocessing practitioners were identified by 50% (*n* = 83) of the respondents as main concerns that need to be addressed in order to increase patient safety in endoscope reprocessing procedures.

**Conclusion:**

This international survey on current flexible endoscope reprocessing identified a large variation for reprocessing practices among different health care facilities/countries. A standardised education and training programme with a competency assessment is essential to prevent reprocessing lapses and improve patient safety.

**Electronic supplementary material:**

The online version of this article (10.1186/s13756-018-0446-6) contains supplementary material, which is available to authorized users.

## Background

Endoscopy related infections represent a threat for healthcare systems worldwide. Recent outbreaks of infections, with highly resistant micro-organisms, have highlighted the problems of contaminated endoscopes, that have intricate mechanisms, multiple internal channels and narrow lumens that are especially difficult to clean [1, 2].

Effective reprocessing of flexible endoscopes involves pre-cleaning, leak testing, cleaning and high-level disinfection followed by rinsing and drying before storage. There are various methods (e.g. microbial cultures, adenosine triphosphate (ATP) test, TOSI^tm^ washer test) to assess the success of the reprocessing process of flexible endoscopes. In addition, multiple guidelines for cleaning and high-level disinfection of flexible endoscopes have been made available by various federal agencies and professional organisations [3–6].

Lapses in the reprocessing process of flexible endoscopes have been associated with several infectious disease outbreaks [1]. Lapses are seen in a range of different types of flexible endoscopes (e.g. duodenoscopes, bronchoscopes, ureteroscopes). In several outbreaks the contamination resulted in patient fatalities [7, 8]. Breaches in the reprocessing of flexible endoscopes were recorded in manual as well as in automated reprocessing [1, 9].

Recently, outbreaks were linked to duodenoscopes, even when strict adherence of the endoscope reprocessing guidelines was followed [10, 11]. The complexity of duodenoscopes is an obstacle for the reprocessing of the device that still needs further research for better solutions.

In light of raised awareness about the necessity for meticulous reprocessing of all types of endoscopes, we present an overview on current practices for endoscope reprocessing in facilities around the world.

## Method

The Infection Prevention and Control (IPC) workgroup within the International Society of Antimicrobial Chemotherapy (ISAC) developed a survey. The survey consisted of 50 questions to assess the current flexible endoscope reprocessing practices worldwide. The survey was designed based on three core elements: stakeholder involvement, assessment of perceived risks and process assessment. An electronic online version of the survey was created using the SurveyMonkey platform and members of the ISAC IPC workgroup were asked to disseminate the survey to other colleagues and healthcare personnel via their network. Other dissemination strategies involved social media communication channels such as Twitter and infection prevention and control blogs. The survey is attached in Additional file 1.

Responses were collected anonymously via SurveyMonkey from June 2015 until May 2016. When identical IP addresses completed the survey more than once, the responses were excluded from the data. As “not-applicable” was one of the possible answers for some of the questions, the number of included responses varied from one question to another.

## Results

A total of 165 completed responses came from 39 different countries. The countries were categorized based on The World Bank’s ranking system. The majority of participants (63%, *n* = 103) were from 22 high-income countries, 31% (*n* = 52) from 12 upper-middle-income countries, and 6% (*n* = 10) from 5 lower-middle-income countries. There were no participants from low-income countries. The participants of the electronic survey were overwhelmingly infection control nurses 63% (n = 103), followed by endoscope nurses 9% (*n* = 15), central sterilization managers 8% (*n* = 14), endoscope unit managers 7% (*n* = 12), decontamination managers 3% (*n* = 5), infectious disease physicians 3% (*n* = 6) and other disciplines 7% (*n* = 12).

### Stakeholders

#### Dedicated endoscope reprocessing professionals

Overall 78% (127/163) of the institutions have a dedicated person to overlook the reprocessing of flexible endoscopes (high-income 84% (86/102) upper-middle-income 71% (36/51) lower-middle-income 50% (5/10)).

#### De-central or central reprocessing of flexible endoscopes

**Fig. 1:**
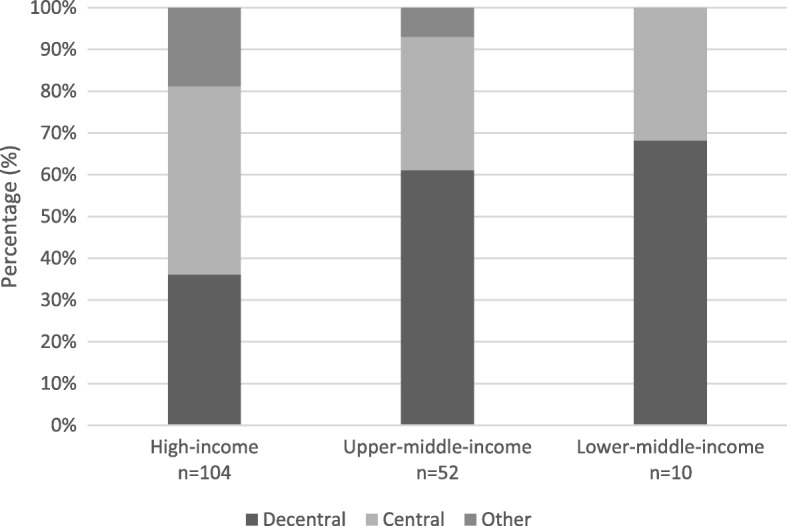
Percentage of facilities with central and decentral reprocessed flexible endoscopes in high- and middle-income countries

Flexible endoscope reprocessing is generally performed in either a central designated area or a de-central (local) area. Higher income countries have more central endoscope reprocessing areas in comparison to upper-middle- and lower-middle-income countries where the majority of the reprocessing is performed de-centrally (Fig. 1). “Other” responses mainly encompassed the operating theatre or the procedure room.

#### Who reprocesses flexible endoscopes

The reprocessing is carried out by different disciplines: reprocessing technicians (50%), dedicated trained endoscopy nurses (38%), trained endoscopy nurses (31%), non-trained endoscopy nurses (9%) and by trained but non-endoscopy related personnel (8%).

### Assessment of perceived risks

#### Ranking of endoscopy reprocessing in relation to patient safety

**Fig. 2:**
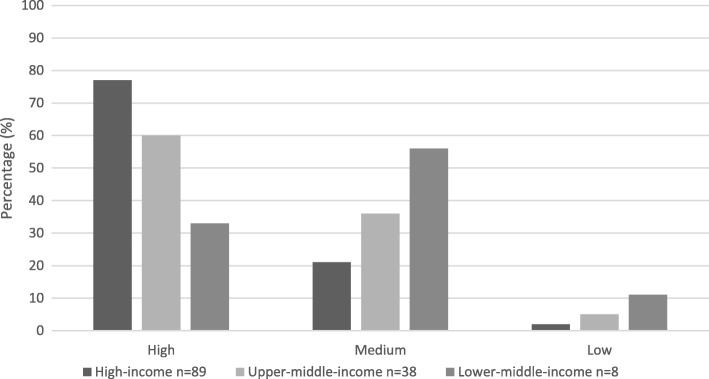
Ranking of the perceived importance in relation to reprocessing flexible endoscopes for patient safety

Professionals in high-income countries categorize endoscopy reprocessing, in relation to patient safety, as a high priority. For upper-middle-income countries a slight decrease in priority was noticeable, and lower-middle-income countries showed a trend to score the patient safety as a medium priority (Fig. 2).

#### Type of flexible endoscopes representing the highest risk

Most of the respondents stated that all flexible endoscopes represent a high risk (45%, 69/155), followed by duodenoscopes (41%, 64/155), bronchoscopes (29%, 45/155) and flexible endoscopes for surgical procedures (27%, 42/155).

#### Outbreak in institutions related to flexible endoscopes

An outbreak situation associated with reprocessing of flexible endoscopes was identified in nearly one fifth (18%) of the participating institutions.

#### Training programs for flexible endoscope reprocessing

**Fig. 3:**
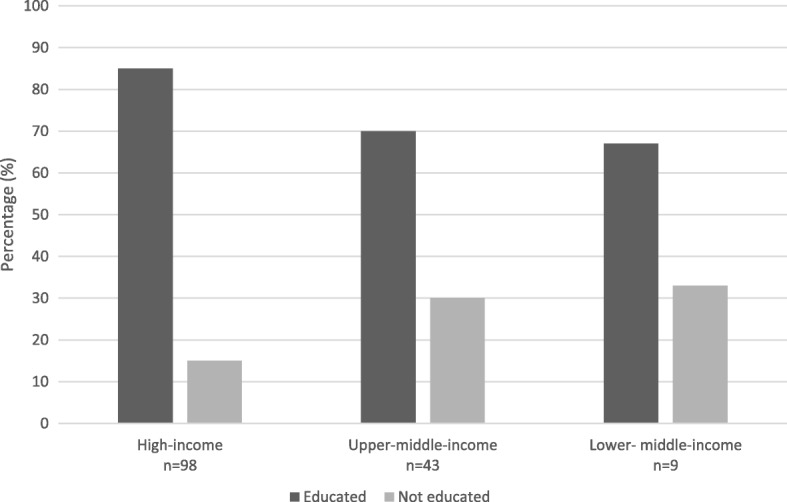
Availability of a training program for endoscope reprocessing staff

Training programs for endoscope reprocessing staff had occurred in 85% of the high-income countries, in 70% of the upper-middle-income countries, and in 67% of the lower-middle-income countries (Fig. 3).

Respondents believed that there was a need to increase the educational level of staff members (33%) and to allocate more resources for endoscopes (33%) to improve the reprocessing process. Respondents identified education as main concern that need to be addressed in order to increase patient safety (50%).

### Process assessment

**Fig. 4:**
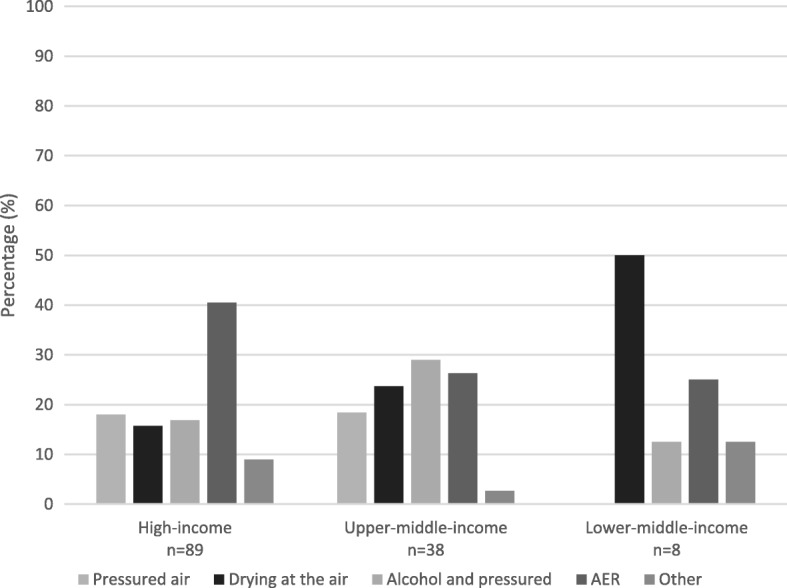
Representation of methods used to dry flexible endoscopes before storage in high- and middle-income countries. AER; Automated Endoscope Reprocessor

Standard operating procedures (SOP) for reprocessing of endoscopes were available in the majority of facilities (Table 1). The different stages in the reprocessing process were not always routinely performed (Table 1). Respondents considered manual cleaning and automatic disinfection most important of all the reprocessing steps taken based on a weighted average (Table 1). Various methods were applied for the drying of flexible endoscopes before storage (Fig. 4). Whereby lower-middle-income countries dry endoscopes predominantly in the air (50%) before storage and most high-income countries dry the endoscopes in the automated endoscope reprocessor (AER) for 40% of the times before storage.

**Table 1:** Routine use, available Standard Operating Procedures (SOP) and importance of flexible endoscope reprocessing steps

Process step	Routine step (%)	SOP (%)	Importance^a^
Bedside flush	74	89	1.24
Manual cleaning	80	90	1.18
Leak testing	77	87	1.36
Manual disinfection	37	54	1.23
Automatic disinfection	79	89	1.18
Drying	57	83	1.44
Drying before storage	77	–	1.33

*SOP* Standard Operating Procedure

^a^Importance was calculated through a weighted average with a range of 1–5

### Process control

A large variation of assessment methods and testing frequencies for the reprocessing of flexible endoscopes was observed (Table 2). Microbial cultures were most often used twice a year for assessment (25%); ATP testing (assessing biological soil level after cleaning and/or disinfection) was used in 41% of the reprocessing facilities.

**Table 2:** Assessment methods and frequency for flexible endoscope reprocessing

Test	Every scope	Once a week	Once a month	Twice a year	Once a year	Never
Microbial culture	9%	6%	18%	25%	11%	31%
TOSI^tm^	23%	10%	14%	6%	9%	38%
Final Rinse water test	15%	10%	22%	15%	10%	28%
Routine ATP	12%	5%	8%	12%	4%	59%
Routine protein test	11%	6%	7%	7%	7%	62%
Routine Other	12%	5%	10%	12%	7%	54%
AER Documentation	63%	5%	3%	6%	4%	19%

*ATP* Adenosine Triphosphate, *AER* Automated Endoscope Reprocessor

The TOSI^tm^ washer test (tests for effectiveness of automated instrument washers) was used in 23% of the facilities for every AER reprocessed endoscope. The final rinse water test was mostly used for monthly assessment (22%). Furthermore, when an AER was used, 63% of the facilities had a process document for each scope.

**Fig. 5:**
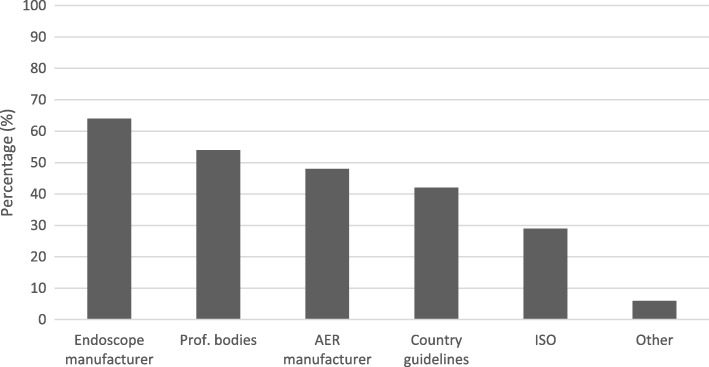
Influence on developed standard operating procedures by the hospital

Instructions provided by the endoscope manufacturer had most influence on the developed standard operating procedures (64%), where ISO standards (29%) and country guidelines (42%) had notably less influence (Fig. 5).

## Discussion

The importance of adequately reprocessing flexible endoscopes has been underestimated for several years, until outbreaks with multidrug resistant organisms (MDRO) in hospitals appeared to have a causal connection to flexible endoscopes [1, 2, 12]. Outbreaks may increase morbidity and mortality, prolong the hospital stay, and lead to secondary transmission to other patients [1]. Mandatory reporting would allow us to estimate the scale at which the problems are occurring and could lead to the issues being addressed adequately.

Recently flexible endoscopes, especially their reprocessing process, have attracted worldwide attention. The Food and Drug Administration (FDA) demanded all three of the manufacturers of endoscopes carry out a post-marketing surveillance study in 2015 to evaluate whether reprocessing guidelines were being followed by the end user [13].

We conducted this survey to evaluate the present state of international endoscope reprocessing in order to better direct future challenges and to give guidance to the global community. There was a decline in perceived importance of flexible endoscope practices in relation to patient safety that fit in line with the economic prosperity ranking system observed.

Reprocessing is performed by different disciplines worldwide, and to date 15–33% of participated facilities in our survey say their staff is still untrained. In order to give equal opportunities worldwide to follow adequate training, freely accessible online training packages for the reprocessing of flexible endoscopes would be helpful and should be offered by endoscope producers or international societies.

Differences were found between central and decentral reprocessing practices in high- and middle-income countries. High-income countries were found to more often have a central reprocessing unit, whereas middle-income countries were found to often have decentral units. The processing of all flexible endoscopes in a central designated area could be considered preferable over the decentral reprocessing, as it has standardised logistics, clear responsibilities, improved quality of process, better quality assurance, efficiency of scale, higher process knowledge, and education standardisation. Decentralized (manual) reprocessing requires the direct commitment of the HCW who thus maintains ownership of the entire process. This likely was the preferred option for participants from lower-middle-income countries, due to the lower cost of (manual) reprocessing.

Outbreaks most often occured through breaches in the reprocessing process, e.g. through defective endoscopes, lacking periodic maintenance [1], or lapses in the manual or automated reprocessing procedures. Lapses often occured through human error, due to a lack of education, missing or unavailable SOP’s, or because the reprocessing staff fail to follow the SOP accurately [1]. In the survey 18% of the participants were aware of an endoscope related outbreak within their facility. Most of the participating facilities in the survey had an SOP available that outlined all the steps involved in the reprocessing process. In contrast, only 57% of the respondents routinely carried out the drying process. The importance of adherence to the procedure described for reprocessing of flexible endoscopes has been confirmed in multiple studies and guidelines [3–6, 14–17]. The assessment of the complete procedure was often subject to the preference of the facility. To date there is no best practice to assess reprocessed flexible endoscopes. As shown in the results, facilities used a variety of different assessment tools. It is of interest that 41% of the facilities used ATP as an assessment method, despite the fact that this is not yet advised in reprocessing guidelines. Consequently, there is an urgent need to determine an optimal method of ensuring endoscopes are free of contamination prior to patient use.

## Conclusion

This international survey on current flexible endoscope reprocessing practices identified the differences in practice and opinion worldwide. The most evident differences were found in the importance of reprocessing with regard to patient safety. Increasing HCWs’ awareness through regular education, with competency assessment and auditing, were found to be important strategies to prevent reprocessing lapses. The differences revealed between countries of high- and middle-income could help in developing guidelines, training programs, and regulations applicable to stakeholders all over the world.

## Additional file

Additional file 1: A survey on current endoscope reprocessing practices. (PDF 398 kb)

## Abbreviations

AER: Automated Endoscope Reprocessor
ATP: Adenosine Triphosphate
FDA: Federal Department Authorization
HAI: Healthcare acquired infections
HCW: Healthcare workers
IPC: Infection Prevention and Control
ISAC: International society Antimicrobial Chemotherapy
MDRO: Multidrug Resistant Organisms
SOP: Standard Operating Procedures
