# Supplementary material for: Worldwide practices on flexible endoscope reprocessing
Source: Antimicrob Resist Infect Control. 2018 Dec 17;7:153. doi: 10.1186/s13756-018-0446-6 (PMC6296091; doi:10.1186/s13756-018-0446-6)
Supplement: Supplementary file 1 — A survey on current endoscope reprocessing practices. (PDF 398 kb) [file 13756_2018_446_MOESM1_ESM.pdf]

A survey on current endoscope reprocessing practices

A survey on current endoscope reprocessing practices

Recent reports of multidrug-resistant infections related to contaminated endoscopes, which have intricate mechanisms and channels that are especially difficult to clean, have raised awareness about the necessity for meticulous reprocessing of all types of endoscopes to prevent the transmission of pathogens to patients.

In response to concerns from various countries about inadequately reprocessed endoscopes and to prevent further transmittal of infections by endoscopes, the ISC Infection Prevention & Control Working Group prioritized this issue in a meeting earlier this year and created a survey on current Endoscope Reprocessing Practices that could be used to compare such practices of institutions around the globe.

This checklist was created by Andreas Voss (Radboud University Nijmegen), other members of ISC IPC working group gave comments and suggestions towards the development of the final survey: (Alex Friedrich, Peter Collignon, Moi Lin Ling, Brenda Ang, Wing Hong Seto, Paul Tambyah, Eli Perencevich, Marin Schweizer, Leanne Frazer, Achilleas Gikas, Tom Gottlieb, Joost Hopman, Nikki Kenters, Inge Huijskens, Kalisvar Marimuthu, Rehab El-Sokkary, Yogandree Ramsamy, Margaret Vos, Ermira Tartari,Debkishore Gupta).

We would ask you to share the link to the on-line survey and encourage as many of your colleagues from various health care facilities to complete this. To complete this survey you need to be involved in Endoscope reprocessing activities or know the guidelines and structure of your institutions with regard to Endoscope reprocessing.

Thank you for your participation!

A survey on current endoscope reprocessing practices

Questionnaire

* 1. In what city and country is your hospital located?

City/Town:

Country:

2. Number of staff members (doctors and nurses) working in the endoscopy unit:

Insert number

3. Average number of patients visiting the endoscopy unit per day:

Insert number

4. What is your Role/Function in the hospital?

- Central Sterilization Service Manager
- Decontamination Manager
- Infection Control Professional/Nurse
- Pharmacist
- Endoscopy Nurse
- Other (please specify):

Stakeholders

5. Does your hospital have a dedicated person who oversees reprocessing of flexible endoscopes?

- Yes
- No

6. Who has overall responsibility for reprocessing of flexible endoscopes in your hospital?

- ◯ Central Sterilization Service Manager
- ◯ Decontamination Manager
- ◯ Head or member of the Infection Control Unit
- ◯ Pharmacist
- ◯ Other (please specify):

7. Where are flexible endoscopes reprocessed in your hospital? (Multiple answers possible)

- ☐ De-central i. Gastroenterology
- ☐ De-central ii. Pneumology
- ☐ De-central iii. Other
- ☐ Central Sterilisation Service
- ☐ Central Endoscope Reprocessing Facility
- ☐ Other (please specify)

8. Who is doing the reprocessing of flexible endoscopes? (Multiple answers possible)

- ☐ Endoscopy Nurse (dedicated and trained)
- ☐ Endoscopy Nurse that is available (all trained)
- ☐ Endoscopy Nurse that is available (no special training)
- ☐ Reprocessing Technician (dedicated, trained in reprocessing)
- ☐ Other (please specify):

A survey on current endoscope reprocessing practices

Assessment of Perceived Risks

9. How does endoscopy reprocessing rank in terms of patient safety priorities compared to other activity in your hospital?

- ◯ High
- ◯ Medium
- ◯ Low

10. What is your view of the level of risk to the patient to develop HAI currently associated with improperly reprocessed endoscopes?

- ◯ High
- ◯ Medium
- ◯ Low

11. What types of flexible endoscopes represent a higher risk to patient related to improper processing? (select all that apply)

- ☐ Flexible endoscopes used for surgical procedures
- ☐ Duodenoscopes
- ☐ Upper GI endoscopes
- ☐ Lower GI endoscopes
- ☐ Bronchoscopes
- ☐ TEE Probe
- ☐ All endoscopes
- ☐ Other (please specify):

12. Did you have an outbreak associated with endoscope reprocessing in your hospital in the past?

- ◯ Yes
- ◯ No

13. Are you aware of outbreaks associated with endoscopes from colleagues in other hospitals?

- ◯ Yes
- ◯ No

14. Do you consider endoscopes to be a source for the transmission of multi-drug resistant micro-organisms, such as Carbapenem resistant enterobacteriaceae (CRE)?

- ◯ Yes
- ◯ No

15. Did you conduct any training programs on flexible endoscope reprocessing?

- ◯ Yes
- ◯ No

A survey on current endoscope reprocessing practices

Process Assessment

A. Bedside Flush: Wipe the outside of the endoscope and flush the channels directly after the patient procedure in the examination room

16. Is this process step routinely performed in your setting?

- ◯ Always
- ◯ Most of the times
- ◯ Sometimes
- ◯ Not

17. Do you have a standard operating procedure (SOP) for this process step?

- ◯ Yes
- ◯ No

18. How important is this process step for the overall reprocessing success?

- ◯ 1 (Very Important)
- ◯ 2
- ◯ 3
- ◯ 4
- ◯ 5 (Unimportant)

19. Is this process step validated in your hospital?

- ◯ Yes
- ◯ No
- ◯ Not applicable

B. Manual cleaning: Manual brushing of the channels and wiping of the outside of the endoscope to clean the endoscope from body fluids, body tissues and other contaminations

20. Do you routinely perform this process step?

- ◯ Always
- ◯ Most of the times
- ◯ Sometimes
- ◯ Not

21. Do you have a standard operating procedure (SOP) for this process step?

- ◯ Yes
- ◯ No

22. How important is this process step for the overall reprocessing success?

- ◯ 1 (Very Important)
- ◯ 2
- ◯ 3
- ◯ 4
- ◯ 5 (Unimportant)

A survey on current endoscope reprocessing practices

C. Leak testing: applying air pressure to the inside of the endoscope insertion tube and then watching for air bubbles identifying leaks

23. Is this process step routinely performed in your setting?

- ◯ Always
- ◯ Most of the times
- ◯ Sometimes
- ◯ Not

24. Do you have a standard operating procedure (SOP) for this process step?

- ◯ Yes
- ◯ No

25. How important is this process step for the overall reprocessing success?

- ◯ 1 (Very Important)
- ◯ 2
- ◯ 3
- ◯ 4
- ◯ 5 (Unimportant)

26. Is this process step validated in your hospital?

- ◯ Yes
- ◯ No
- ◯ Not applicable

International Society of Chemotherapy for Infection and Cancer

A survey on current endoscope reprocessing practices

D. Manual Disinfection: Manual disinfection to remove any viable bacterial or viral contamination of the endoscope without the help of an automated reprocessor (AER)

27. Do you routinely perform this process step?

- Yes, always
- Yes, sometimes
- No (If answer is “No” please move ahead to question 32).

28. Do you have a standard operating procedure (SOP) for this process step?

- Yes
- No

29. How important is this process step for the overall reprocessing success?

- 1 (Very Important)
- 2
- 3
- 4
- 5 (Unimportant)

A survey on current endoscope reprocessing practices

E. Automatic Disinfection: Automated disinfection to remove any viable bacterial or viral contamination of the endoscope with the help of an automated reprocessor (AER). The AER may or may not have an automated cleaning cycle prior to disinfection cycle.

30. Do you routinely perform this process step?

- Yes, always
- Yes, sometimes
- No

31. Do you have a standard operating procedure (SOP) for this process step?

- Yes
- No

32. How important is this process step for the overall reprocessing success?

- 1 (Very Important)
- 2
- 3
- 4
- 5 (Unimportant)

33. Is this process step validated in your hospital?

- Yes
- No
- Not applicable

34. Do you use dedicated strips for the evaluation of the effective concentration of the disinfectant solution?

- Yes
- No

A survey on current endoscope reprocessing practices

F. Drying: Drying of the endoscope prior to immediate patient use without overnight storage of the endoscope

35. Do you routinely perform this process step?

- Yes, always
- Yes, most of the times (on exception “wet” use)
- No

36. How do you dry the endoscope before patient use?

- ◯ Pressured air
- ◯ Drying at the Air
- ◯ Alcohol Flush and Pressurised Air
- ◯ Drying Cycle in an AER
- ◯ Other (please specify)

37. Do you have a standard operating procedure (SOP) for this process step?

- ◯ Yes
- ◯ No

38. How important is this process step for the overall reprocessing success?

- ◯ 1 (Very Important)
- ◯ 2
- ◯ 3
- ◯ 4
- ◯ 5 (Unimportant)

39. Is this process step validated in your hospital?

- ◯ Yes
- ◯ No
- ◯ Not applicable

G. Drying before Storage/Drying and Storage: Drying of the endoscope in storage or before the endoscope is put into storage

40. Do you routinely perform this process step?

- ◯ Yes, always
- ◯ Yes, most of the time
- ◯ No

41. How do you dry the endoscope before storage?

- ☐ Pressured air
- ☐ Drying at the Air
- ☐ Alcohol Flush and Pressurised Air
- ☐ Drying Cycle in an AER
- ☐ Drying in the storage chamber
- ☐ Other (please specify)

42. How important is this process step for the overall reprocessing success?

- ◯ 1 (Very Important)
- ◯ 2
- ◯ 3
- ◯ 4
- ◯ 5 (Unimportant)

43. Is this process step validated in your hospital?

- ◯ Yes
- ◯ No
- ◯ Not applicable

Process Control

44. What influences your local/hospital SOPs most?

- [ ] Country Guidelines
- [ ] Endoscope Manufacturer Instructions
- [ ] AER Manufacturer Instructions
- [ ] ISO Standard
- [ ] Professional Bodies Guidelines
- [ ] Other (please specify)

45. What can further increase patient safety in endoscope reprocessing? (PLEASE RANK THEM 1-4)

- Education
- Improved Quality Control
- Monitoring of Reprocessing
- Sterilization of Endoscopes

46. What processes or checks or measures do you have in place to ensure the cleaning and high level disinfection protocol/ procedures are being correctly followed? (check all that apply)

	every scope	once a week	once a month	2-6 month	7-12 month	never
Endoscope Microbial Culture	○	○	○	○	○	○
Inline AER Monitoring Test (e.g TOSI)	○	○	○	○	○	○
Final Rinse Water Test	○	○	○	○	○	○
Routine Endoscope monitoring i. ATP	○	○	○	○	○	○
Routine Endoscope monitoring ii. Protein	○	○	○	○	○	○
Routine Endoscope monitoring iii. Other	○	○	○	○	○	○
AER process documentation (Print out)	○	○	○	○	○	○
Audits	○	○	○	○	○	○
Other	○	○	○	○	○	○

(please specify):

47. Is there strict regulations with regards to the time elapsed between endoscopy procedures?

- ○ Yes
- ○ No

48. What is the most influencing constraint:

- ○ Financial
- ○ Managerial
- ○ Educational
- ○ None

49. What do you need to improve the process (more than one choice):

- [ ] Availability of a final checklist on the different steps of reprocessing
- [ ] Increase the awareness of managers
- [ ] Raise the educational level of staff members.
- [ ] Allocate more resources for endoscopy units.
- [ ] Apply strict regulations as regards the implementation of the process.
- [ ] All of the above
- [ ] None

50. Number of flexible endoscopy units in your hospital:

Insert Number: ____________________
